# The relationship between the ages and stages questionnaire, 3rd edition scores in early childhood and future cognitive abilities in young Nepalese children

**DOI:** 10.1186/s12887-024-05112-3

**Published:** 2024-10-10

**Authors:** Merina Shrestha, Ingrid Kvestad, Mari Hysing, Suman Ranjitkar, Manjeswori Ulak, Ram K. Chandyo, Tor A. Strand

**Affiliations:** 1 Department of Pediatrics, Institute of Medicine, Tribhuvan University, Kathmandu, Nepal; 2 Regional Centre for Child and Youth Mental Health and Child Welfare, NORCE Norwegian Research Centre, Bergen, Norway; 3 Department of Research, Innlandet Hospital Trust, Lillehammer, Norway; 4 Department of Psychosocial Science, Faculty of Psychology, University of Bergen, Bergen, Norway; 5 Centre for International Health, Department of Global Public Health and Primary Care, University of Bergen, Bergen, Norway; 6 Department of Community Medicine, Kathmandu Medical College, Kathmandu, Nepal

**Keywords:** Age and stage questionnaire, Correlation, WPSSI, Intelligence

## Abstract

**Background:**

The Ages and Stages Questionnaire 3rd edition (ASQ-3) could be a feasible tool in resource-poor settings. Little is known on the relationship between scores on the ASQ-3 and later intellectual abilities in these settings.

**Aims:**

To examine the relationship between ASQ-3 scores during the first and second year of life and intellectual abilities at 4 years of age in Nepalese children.

**Methods:**

In a cohort of 600 children at-risk of stunting, the ASQ-3 was performed at 6–11 and 18–23 months, and the Wechsler Preschool and Primary Scales of Intelligence, fourth edition (WPPSI-IV) at 4 years. We examined the relationship between the ASQ-3 scores and WPPSI-IV full scale IQ (FSIQ) using Spearman correlation coefficients and linear regression models.

**Results:**

Correlations between ASQ-3 total scores and FSIQ was 0.17 (95% CI 0.07, 0.27) at 6–11 and 0.34 (95% CI 0.26, 0.44) at 18–23 months explaining 2 and 12% of the variance respectively. Except for the communication subscale at 18–23 months with moderate correlations, correlations between the ASQ-3 subscales and FSIQ were weak.

**Conclusion:**

Our findings suggest a modest relationship between ASQ-3 scores in early childhood and intellectual abilities at 4 years.

**Supplementary Information:**

The online version contains supplementary material available at 10.1186/s12887-024-05112-3.

## Introduction

About 43% of children below five years of age in low and middle-income countries (LMIC) are at risk of developmental delay [1]. In Nepal, an estimated 42% of children between 3 and 4 years of age experience poor cognitive and/or socio-emotional development when measured by an early childhood development index [2]. To track early child development, we need reliable and valid measures. While the Bayley Scales of Infant and Toddler Development (BSID) is often considered the gold standard, this tool may not be feasible in resource-limited settings. In this context, the Ages and Stages Questionnaire 3rd Edition (ASQ-3), widely utilized for developmental screening, has been recommended as a potential effective tool for the early detection of childhood disabilities [3]. In Nepal, ASQ-3 has already been used in several community-based studies with acceptable feasibility [4, 5].

The relationship between the ASQ and other measures of early child development has been addressed in several systematic reviews and meta-analyses, both through addressing concurrent (the degree to which an instrument correlate with another instrument measured at the same time) and predictive (the degree to which an instrument predicts future performance) validity. Results from a recent systematic review and meta-analysis suggest that ASQ-scores 2 standard deviations below the mean had moderate sensitivity and specificity to predict developmental delays in children 12 to 60 months of age [6]. In another meta-analysis on developmental assessment tools, ASQ-3 was the most frequently used tool showing an overall adequate diagnostic accuracy of the developmental assessment tools [7]. A third recent systematic review examined the predictive validity of parent-completed developmental questionnaires on long-term cognitive achievement and/or school performance. This review found that the ASQ was the most frequently used developmental screening questionnaire with 32 studies (10 cohorts), and that the questionnaire showed adequate capacity to predict later cognitive achievement/school performance in diverse social, economic and cultural settings [8].

Many studies have specifically investigated the screening properties for infants who are at high risk, with most studies focusing on infants born prematurely. For instance, in a study of prematurely born toddlers, the ASQ-3 satisfactorily identified the BSID Development Quotient (DQ) of less than 85 at 2 years [9]. Similarly, ASQ-3 showed adequate psychometric properties and modest agreement with BSID in prematurely born children at 8, 18, and 30 months of age [10]. Moreover, when children born before 35 weeks of gestation were assessed with the ASQ at 18, 24 and 36 months, the predictive ability for severe school difficulties at 5 years was adequate [11].

Most of the aforementioned studies are from high-income settings, and the relationship between early ASQ-scores and later cognition in LMIC such as Nepal is unknown. Further, most of the studies on high-risk infants have focused on premature infants and in LMIC there might be other relevant high-risk groups. In Nepal, 25% of children below five years of age are stunted [12], and stunting has been suggested as one of the key risk factors for developmental delay in LMICs [13]. This is also demonstrated by a study in India showing a linear relationship between length-for-age z score (LAZ) scores below − 2 and ASQ scores in young children [14].

In a sample of Nepalese children at risk of stunting during infancy, we measured ASQ-3 at two timepoints during early childhood and cognitive ability when the children were approximately 4 years. We have previously shown that the ASQ-3 scores from infants 6–11 months old discriminate between children with various risks of poor development such as birth weight, linear growth and maternal educational level supporting its construct validity in this setting [4]. The aim of the current study was to examine the relationship between scores on the ASQ-3 during the first and second year or life and cognitive abilities measured by the Wechsler Preschool and Primary Scales of Intelligence fourth edition (WPPSI-IV) when the children were approximately 4 years.

## Methods

### Sample

Data in these analyses stem from a cohort of Nepalese children originally participating in a community-based randomized double-blind placebo-controlled nutrition trial (ClinicalTrials.gov: NCT02272842) [15]. In the original trial, conducted in Bhaktapur, Nepal, 600 infants, 6 to 11 months of age at risk of stunting, were recruited from April 2015 until February 2017. Half of them received vitamin B12 daily for 12 months, and the results showed no effect of the supplementation on the primary outcomes; neurodevelopment (as measured by the BSID-III), growth and hemoglobin [15]. Inclusion criteria were risk of stunting (length-for-age z-score (LAZ) < 1 SD), living in a family planning to reside in the area for the next 12 months, and where parents consented for participation. Children with severe systemic illness requiring hospitalization, severe malnutrition (weight‐for‐length z‐score ≤ 3 SD) and with severe anemia (Hb < 7 g/dl) were excluded from the study.

After inclusion in the original trial, children were invited for follow-up assessments every 12 months. Of the 600 enrolled children, 592 (98.6%) completed the ASQ-3 assessment at 6–11 months (T1) and 509 (84.8%) at 18–24 months (T2). At approximately 4 years (T3), 533 (88.8%) of the enrolled children completed the WPPSI-IV.

### Procedure

Children aged 6 to 11 months were recruited from the immunization clinic or through door-to-door visits, as identified by field workers (FWs). The length of the children was measured using portable infantometers (Seca, Hamburg, Germany), and those whose LAZ was less than 1 SD, as confirmed by a supervisor or physician were enrolled in the study. Stunting was defined as LAZ < − 2.

Demographic information of the child and families, including parental age, educational level and occupation, and type of family, was obtained from the mothers at the time of enrollment. Birth weight was obtained from the hospital record file, Families were categorized as nuclear or joint family in which a nuclear family includes parents and their children, and joint family includes all the extended family members like grandparents, uncles and aunts living together in a same house and sharing the same kitchen. The educational level was categorized into illiterate, primary school, secondary school, School Leaving Certificate/intermediate school, bachelor’s degree, and above. We categorized the occupation of mothers and fathers as no formal work, agriculture, carpet worker, daily wage earner, self-employed, working in the service, and working abroad.

### Developmental assessments

#### ASQ-3

ASQ-3 is a developmental screening tool that includes five developmental domains: communication, gross motor, fine motor, problem solving, and personal social skills. Each subscale consists of 6 items coded yes = 10, sometimes = 5, and not yet = 0. The subscale and total scores are the summation of item scores in each subscale and of the total test. Subscale scores range from 0 to 60 while the total score range from 0 to 300. For missing items, the mean score of the individual on the subscale was imputed as recommended in the ASQ-3 manual [16].

The questionnaires were translated to Nepali following official recommendations for standards in translation and adaptations [17]. The first author, a developmental pediatrician, translated the questionnaires from English to Nepali. The back translation was done by a Nepalese professor in English literature, not otherwise involved in the study. Finally, the back translation was reviewed by the first author and a Norwegian psychologist and compared with the original version. The final version of the questionnaires was adapted after several discussions amongst the psychologists and fieldworkers. A few adjustments were made during piloting of the questionnaires. As children did not like soft dolls, we replaced it with a plastic doll. As strollers were not available, we custom-made a wooden stroller with wheels and handle.

We used questionnaires for 6, 8, 9, 10 and 12 months at baseline and 18, 20, 22 and 24 months for the second follow up. Three experienced FWs with education levels above grade 10 and at least 15 years of experience in research fieldwork were identified to conduct the assessments. They had primarily been involved in maternal interviews for a nutrition research project and were well-trained in anthropometric assessments of children. Since they were local residents, they had good rapport with the residents of the community. The training on ASQ assessment was conducted in Nepali language by the first author herself. Throughout the training, the children’s activities were directly monitored as they played with toys, occasionally with the facilitators personally demonstrating the play with various toys to guide scoring. Standardization exercises were performed on 20 children after training using a developmental pediatrician (first author) as the gold standard [4]. The intraclass correlations (ICC) from these exercises reached 0.88 for the total ASQ-3 score and ranged from 0.71 to 0.92 for the subscales. During the study period, 3% of the assessments were double scored by the first author reaching an ICC for the total ASQ-3 score of 0.52 and ICCs ranging from 0.36 to 0.63 for the subscales.

An adapted version of the ASQ *“home procedure”* was used in the current study [4, 16]. In this procedure, FWs directly observed the children and filled in the questionnaires. When the performance could not be directly observed during the assessment, the mothers/caretakers were asked about the child’s abilities, and scores were based on reports from the mother/caretaker. Children were assessed at 2 timepoints; 6–11 months and one year after when the children were between 18 and 23 months. Most of the assessments were performed at the child`s homes and a few were done at the study clinic when it was not feasible to perform them in their homes.

#### Wechsler preschool and primary scale of intelligence-IV

The Wechsler Preschool and Primary Scale of Intelligence-IV (WPPSI-IV) is a test of general abilities designed for children between 2 years and 6 months to 7 years and 7 months [18]. Six subtests, namely Information, Receptive Vocabulary, Block Design, Picture Memory, Object Assembly and Zoo Locations were included to generate a Full-Scale IQ (FSIQ), the Verbal comprehension index (VCI), the Visuospatial index (VSI), and the Working memory index (WMI). The FSIQ and the index scores (all with an expected mean (SD) of 100 (15)) were calculated based on US norms [18, 19].

An experienced senior psychologist trained three psychologists on the application of WPPSI-IV. Prior to the study, standardization exercises were performed in 20 children with the requirement to reach excellent agreement with an expert rater (ICC > 0.90). Cultural adaptation was not required for WPPSI-IV. During the study period, 10% of the assessments were double scored by the senior psychologist in a randomized manner, reaching an ICC > 0.98. All WPPSI-IV assessments were performed at the study clinic in designated rooms.

### Statistical analyses

Data are presented as means (SD) or numbers (%). Spearman correlation was used to examine the correlation between ASQ-3 total and subscale score at the two timepoints (6–11 months and 18–23 months) and WPSSI FSIQ and index scores. For the interpretation of results, correlations 0.0-0.3 were considered negligible, 0.3–0.5 as low positive; 0.5–0.7 moderate positive; 0.7–0.9 high positive, and 0.9 to 1.0 as very high positive correlation [20]. The associations between the total ASQ-3 scores at both timepoints and the WPPSI FSIQ are depicted in fitted regression lines. We also calculated the R^2^ using linear regression analysis with the ASQ-3 subscale and total score at the two timepoints as independent variables and the FSIQ as the dependent variable.

## Results

Baseline demographic and clinical characteristics of the 600 children and parents at the time of enrolment are shown in Table 1. The mean (SD) age of children at enrolment was 8.4 (1.8) months and 48.5% were female. The mean birth weight (SD) was 3040.1 (1413.4) grams, and 162 (27.1%) children were stunted. The baseline characteristics are similar for the 600 infants and parents in the original study and those in the longitudinal samples (see supplementary Table 1). The mean (SD) age of the children (*n* = 509) at the first follow up (T2) was 20.0 (1.8) months and at the second follow up (T3, *n* = 533) 43.9 (1.9) months.

**Table 1:** Baseline demographic characteristics at of a Nepalese child cohort

**Child characteristics**		
Age in months, mean (sd)		8.4 (1.8)
Female		291 (48.5%)
Birth weight in grams, mean (sd)		3040.1 (1413.4)
Children stunted at T1		162 (27.1%)
**Family characteristics**		
Living in joint family		292 (48.7%)
Maternal age in years at T1, mean (sd)		27(4)
Maternal literacy		
Illiterate up to grade 5		223 (37.2)
Grade 5 to High School		261 (43.5)
Bachelor and above		116 (19.3)
Maternal occupation		
No work/Agriculture		373 (62.2)
Daily wage earner		90(15)
Service/self employed		137 (22.8)
Paternal literacy		
Illiterate up to grade 5		212 (35.3)
Grade 5 to high School		280 (46.7)
Bachelor and above		108 (18)
Paternal occupation		
No Work/Agriculture		34 (5.7)
Daily wage earner		240 (40)
Service/self employed		326 (54.3)

All figures are numbers (%) if not otherwise specified

**Fig. 1:**
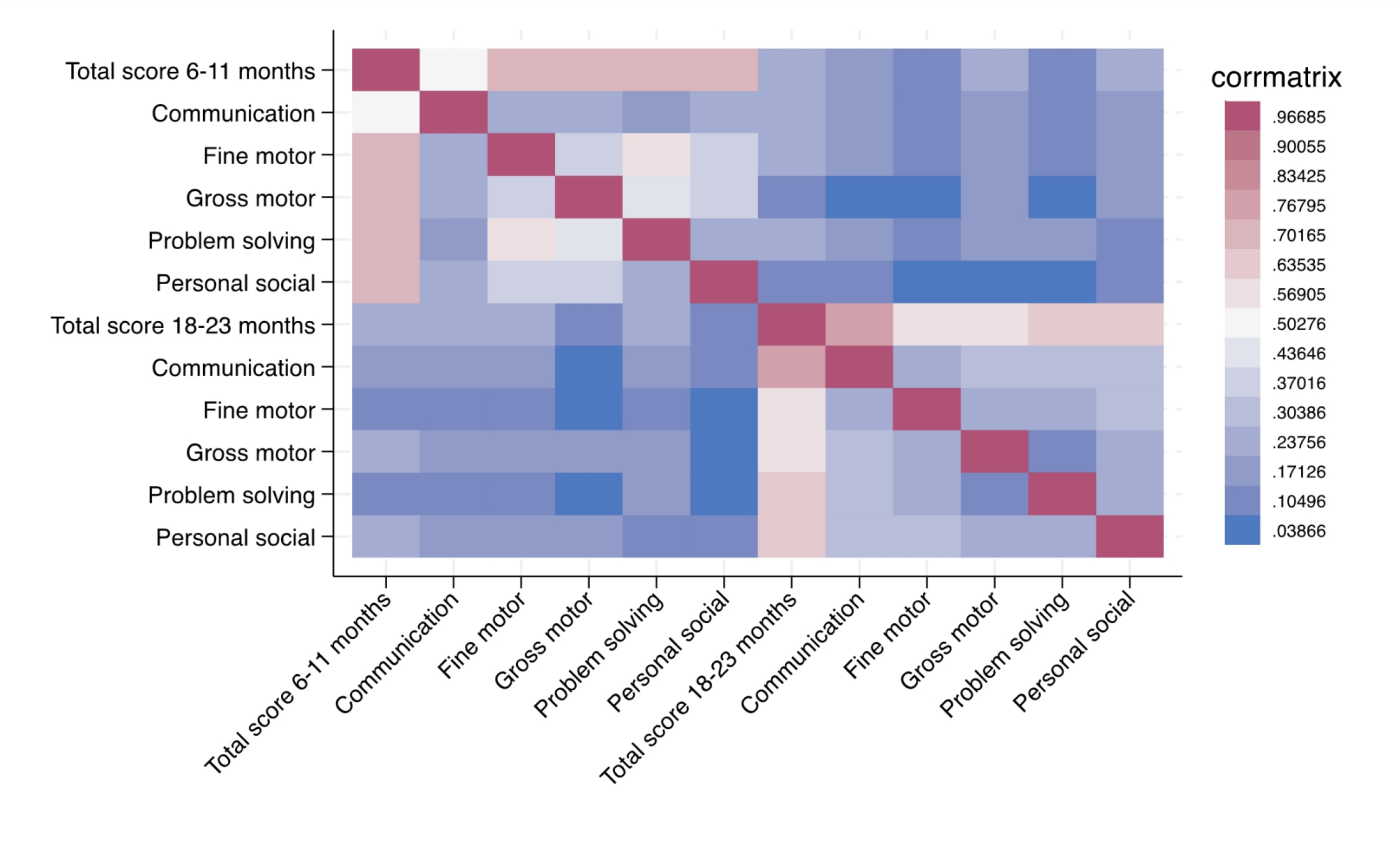
Correlations between ASQ-3 subscale and total score at 6–11 and 18–23 months in a Nepalese child cohort (*N* = 539)

The mean (SD) ASQ-3 total score at 6–11 months and 18–23 months were 209.8 (39.01) and 211.4 (32.8), respectively. At 42–47 months, the mean FSIQ was 84.7 (8.6). The correlations between the ASQ total and subscale scores at 6–11 months and 18–23 months are shown in a heat map in Fig. 1. Correlations between the total and subscale scores within the same age range were low to moderate positive, while correlations between subscale scores within the same age range were low positive. Correlations between total and subscale scores between the two age ranges were negligible.

Correlations between the ASQ-3 total and subscale scores at 6–11 months and 18–23 months and the WPSSI-IV FSIQ and index scores at 42–47 months are shown in Table 2. The correlation coefficient between the ASQ-3 total score at 6–11 months and FSIQ was 0.17 (95% CI 0.07, 0.27) and 0.34 (95% CI 0.26, 0.44) at 18–23 months. The correlation between the ASQ subscales and FSIQ ranged from 0.07 (CI 95% -0.01, 0.16) to 0.19 (CI 95% 0.10,0.27) at 6–11 months and 0.16 (95% CI 0.08, 0.24) to 0.32 (95% CI 0.24, 0.39) at 18–23 months. All the correlations were weak, except for the communication subscale at 18–23 months that showed a moderate correlation with the FSIQ at 42–47 months (0.32 (95% CI 0.24, 0.39).

**Table 2:** Correlation matrix between total and subscale ASQ-3 score at 6–11 and 18–23 months and WPPSI full scale IQ and index scores at 42–47 months in a Nepalese child cohort

ASQ-3		Total score	Communication	Gross motor	Fine motor	Problem solving	Personal social
WPPSI-IV	*N*	Spearman`s Rho 95% CI	Spearman`s Rho 95% CI	Spearman`s Rho 95% CI	Spearman`s Rho 95% CI	Spearman`s Rho 95% CI	Spearman`s Rho 95% CI
6–11 months	526						
Full IQ score		0.17 (0.07, 0.27)	0.19 (0.10, 0.27)	0.12 (0.04, 0.21)	0.13 (0.03, 0.22)	0.07 (-0.01, 0.16)	0.09 (-0.01, 0.17)
Verbal comprehension		0.17 (0.08, 0.27)	0.21 (0.13, 0.30)	0.16 (0.08, 0.25)	0.08 (-0.01, 0.16)	0.06 (-0.03, 0.15)	0.09 (0.00, 0.18)
Visuo-spatial		0.10 (-0.00, 0.21)	0.09 (0.01, 0.17)	0.06 (-0.03, 0.15)	0.11 (0.01, 0.21)	0.04 (-0.06, 0.14)	0.06 (-0.03, 0.14)
Working memory		0.14 (0.04, 0.25)	0.10 (0.02, 0.17)	0.07 (-0.01, 0.16)	0.14 (0.04, 0.25)	0.09 (-0.00, 0.19)	0.08 (-0.01, 0.18)
18–23 months	509						
Full IQ score		0.34 (0.26, 0.44)	0.32 (0.24, 0.39)	0.19 (0.08, 0.29)	0.17 (0.09, 0.26)	0.16 (0.08, 0.24)	0.20 (0.12, 0.28)
Verbal comprehension		0.28 (0.18, 0.38)	0.24 (0.15, 0.33)	0.13 (0.02, 0.24)	0.14 (0.04, 0.23)	0.15 (0.06, 0.24)	0.08 (0.10, 0.27)
Visuo-spatial		0.27 (0.18, 0.36)	0.24 (0.16, 0.32)	0.16 (0.06, 0.27)	0.16 (0.08, 0.24)	0.10 (0.00, 0.19)	0.18 (0.09, 0.27)
Working memory		0.27 (0.17, 0.37)	0.25 (0.17, 0.33)	0.18 (0.08, 0.28)	0.15 (0.05, 0.25)	0.11 (0.03, 0.20)	0.13 (0.04, 0.22)

**Fig. 2:**
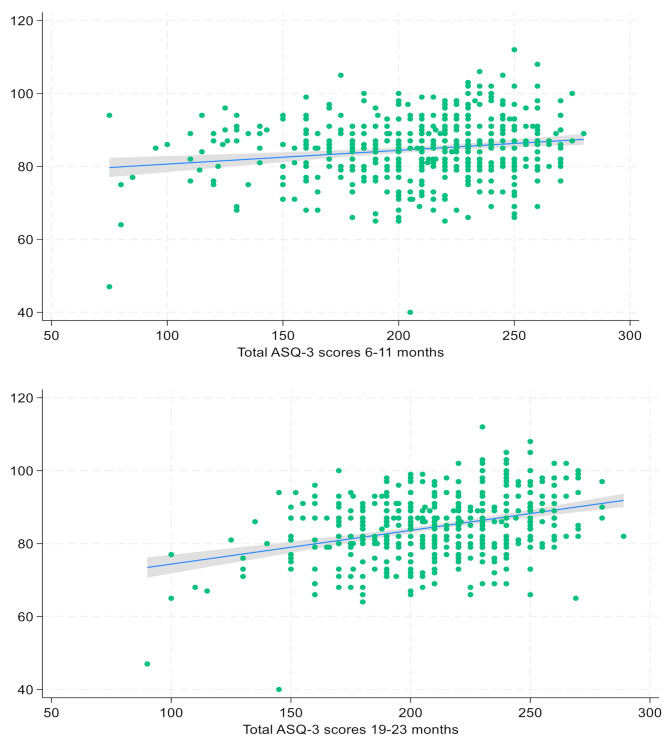
Fitted regression lines between total ASQ-3 scores at 6–11 and 18–23 months and WPPSI full scale IQ at approximately 4 years in a Nepalese child cohort

**Fig. 3:**
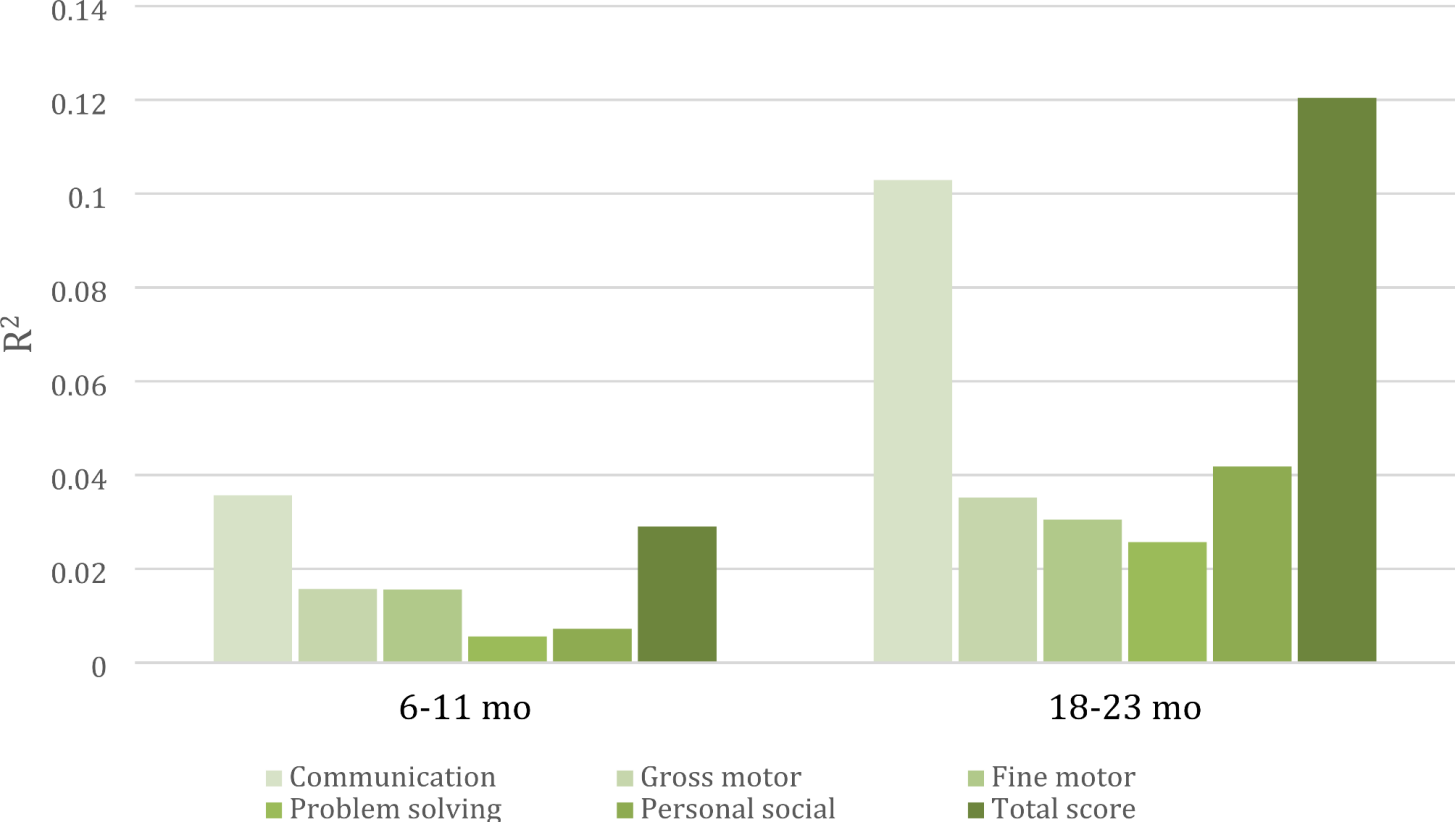
R^2^ of the total and subscale scores at 6–11 and 18–23 months on the WPPSI full scale IQ at approximately 4 years in a Nepalese child cohort

The associations between the total ASQ-3 scores at 6–11 months and 18–23 months and the WPPSI FSIQ are shown in fitted regression lines in Fig. 2. While the total ASQ-3 score explains 3% (R^2^ = 0.03) of the variance in the FSIQ, the total ASQ-3 score at 18–23 months explained 12% (R^2^ = 0.12). The R^2^ for subscales and total scores at both timepoints are shown in Fig. 3. While the scores explain less than 4% (R^2^ = 0.036) at 6–11 months, the communication subscale at 18–23 months explains approximately 10% (R^2^ = 0.103).

## Discussion

The current study was conducted to examine the relationship between ASQ-3 measures in early childhood and cognitive abilities at 4 years in Nepalese children. The study found weak correlations between the ASQ-3 total score at 6–11 months and 18–23 months and FSIQ at 4 years.

Being from a LMIC setting, infants in the current study are characterized by a unique risk profile beyond that of premature born infants. Few studies in the previous meta-analyses are from LMIC, hence, results from the current study are not directly comparable to the previous ones.

Although with overlapping CIs, correlation coefficients indicate stronger correlation between scores at 18–23 months and the FSIQ at 4 years, than between scores taken when the infants were 6–11 months. This is underlined by the explained variance of only 3% from scores when the children were 6–11 months compared to 12% when the children were 18–23 months. Other studies have shown better predictive abilities of the ASQ scores when the children are older on severe school difficulties at 5 years of age [11]. When Chilian children aged 8, 18 and 30 months was assessed with the ASQ, ASQ scores at 30 months had better predictive ability for WISC-III scores at school age [21]. In a previous study from the same cohort as the current one, BSID scores showed negligible correlation (0.10 (95%CI 0.01, 0.18)) at 6–11 months and gradually improved (0.52 (95%CI 0.45, 0.58)) to 30–35 months [19]. The current results, in conjunction with other studies suggest that screening at later age better predict cognitive abilities. Nevertheless, even though developmental assessments at a younger age are poor predictors of future cognitive abilities, these assessments may be helpful for identifying children at risk who require interventions.

Interestingly, while all subscales showed weak correlations with the FSIQ at 42–47 months, the communication subscale at 18–23 months was moderately correlated with the FSIQ. Other studies have also found language skills at young age to better predict IQ at 4.5 years old even after controlling for socioeconomic status, gestational age and medical complications [22, 23]. Language skills in younger age does not only predict cognition at school age but can also predict adult literacy [24]. A 29 year long longitudinal study in the US general population showed that early receptive language problems were associated with poor adult literacy. Language development requires attention to sounds of speech, memory of new vocabulary, and problem solving with integrating information from different sources. This skill being a key factor for later critical thinking and problem solving could be a reason for the strong predictive ability of IQ in children [25]. In the same cohort the predictive ability of the language composite score of the BSID, was acceptable and good using IQ < 70 and < -2SD below the sample mean at 6-11months and 30–35 months, respectively. Hence, findings suggest early language development could be a key indicator for later cognition.

The study has several strengths, including a large sample size and high-quality outcome assessments with well-trained FWs for the ASQ assessments and psychologists for assessing cognitive abilities. The retention rate of children at the 4-year follow up was also high (88.8%). The current study used a *home procedure* which differs from the questionnaire-based approach in many of the previous studies. Further, while the inter-rater reliability was high during training, there was some assessor drift with poorer ICCs throughout the study period [4].

## Conclusion

This study in a LMIC setting found weak correlations between Ages and Stages Questionnaire (ASQ-3) scores in early childhood and cognitive abilities at 4 years. The results underscore the importance of assessing developmental milestones at later ages for better predictive abilities, at the same time, results suggest the significance of early language development as a key indicator for later cognition.

## Electronic supplementary material

Below is the link to the electronic supplementary material.

Supplementary Material 1

## Data Availability

To meet ethical requirements for the use of confidential patient data, requests must be approved by the Nepal Health Research Council (NHRC) and the Regional Committee for Medical and Health Research Ethics in Norway. Requests for data should be sent to the authors, by contacting Child Health Research Project, Department of Child Health, Institute of Medicine, Tribhuvan University (chrp2015@gmail.com), or by contacting the Department of Global Health and Primary Care at the University of Bergen (post@igs.uib.no).

## Code availability

STATA 18 (StataCorp, College Station, Texas, USA)

## References

[1] Lu C, Black MM, Richter LM. Risk of poor development in young children in low-income and middle-income countries: an estimation and analysis at the global, regional, and country level. Lancet Global Health. 2016;4(12):e916–22. 27717632 10.1016/S2214-109X(16)30266-2 PMC5881401

[2] McCoy DC, et al. Early childhood developmental status in low-and middle-income countries: national, regional, and global prevalence estimates using predictive modeling. PLoS Med. 2016;13(6):e1002034. 27270467 10.1371/journal.pmed.1002034 PMC4896459

[3] Singh A, Yeh CJ, Blanchard SB. Ages and stages questionnaire: a global screening scale. Boletín Médico Del Hospital Infantil de México. (English Edition). 2017;74(1):5–12. 10.1016/j.bmhimx.2016.07.008 29364814

[4] Shrestha M, et al. The feasibility of the A ges and S tages Q uestionnaire for the assessment of child development in a community setting in Nepal. Child Care Health Dev. 2019;45(3):394–402. 30818415 10.1111/cch.12654

[5] Thorne-Lyman AL, et al. Dietary diversity and child development in the far west of Nepal: a cohort study. Nutrients. 2019;11(8):1799. 31382653 10.3390/nu11081799 PMC6722734

[6] Muthusamy S et al. Utility of the ages and stages questionnaire to identify developmental delay in children aged 12 to 60 months: a systematic review and meta-analysis. JAMA Pediatr. 2022 Oct 1;176(10):980–89 10.1001/jamapediatrics.2022.3079 PMC9425289 36036913

[7] Rah SS, et al. Systematic review and meta-analysis: real-world accuracy of children’s developmental screening tests. J Am Acad Child Adolesc Psychiatry. 2022. 10.1016/j.jaac.2022.12.014 36592715

[8] Schonhaut L et al. Predictive validity of developmental screening questionnaires for identifying children with later cognitive or educational difficulties: a systematic review. Front Pead. 2021;9. 10.3389/fped.2021.698549 PMC8651980 34900855

[9] Flamant C, et al. Parent-completed developmental screening in premature children: a valid tool for follow-up programs. PLoS ONE. 2011;6(5):e20004. 21637833 10.1371/journal.pone.0020004 PMC3102669

[10] Schonhaut L, et al. Validity of the ages and stages questionnaires in term and preterm infants. Pediatrics. 2013;131(5):e1468–74. 23629619 10.1542/peds.2012-3313

[11] Halbwachs M, et al. Predictive value of the parent-completed ASQ for school difficulties in preterm-born children. Neonatology. 2014;106(4):311–6. 25198520 10.1159/000363216

[12] Nepal M. o.H.a.P.o., Nepal demographic and health survey. 2022.

[13] Perkins JM, et al. Understanding the association between stunting and child development in low-and middle-income countries: next steps for research and intervention. Soc Sci Med. 2017;193:101–9. 10.1016/j.socscimed.2017.09.039 29028557

[14] Kvestad I, et al. Diarrhea, stimulation and growth predict neurodevelopment in young north Indian children. PLoS ONE. 2015;10(3):e0121743. 25826376 10.1371/journal.pone.0121743 PMC4380317

[15] Strand TA, et al. Effects of vitamin B12 supplementation on neurodevelopment and growth in Nepalese infants: a randomized controlled trial. PLoS Med. 2020;17(12):e1003430. 33259482 10.1371/journal.pmed.1003430 PMC7707571

[16] Squires J, Bricker DD, Twombly E. Ages & stages questionnaires. Paul H. Brookes Baltimore; 2009.

[17] Wild D, et al. Principles of good practice for the translation and cultural adaptation process for patient-reported outcomes (PRO) measures: report of the ISPOR task force for translation and cultural adaptation. Value Health. 2005;8(2):94–104. 15804318 10.1111/j.1524-4733.2005.04054.x

[18] Wechsler D. Wechsler preschool and primary scale of intelligence—fourth edition. TX: The Psychological Corporation San Antonio; 2012.

[19] Kvestad I, et al. The stability of the Bayley scales in early childhood and its relationship with future intellectual abilities in a low to middle income country. Early Hum Dev. 2022;170:105610. 35728398 10.1016/j.earlhumdev.2022.105610

[20] Hinkle DE, Wiersma W, Jurs SG. Applied statistics for the behavioral sciences. Volume 663. Houghton Mifflin college division; 2003.

[21] Schonhaut L, et al. Comparison between ages & stages questionnaire and Bayley scales, to predict cognitive delay in school age. Early Hum Dev. 2020;141:104933. 31775095 10.1016/j.earlhumdev.2019.104933

[22] Bogičević L, Verhoeven M, van Baar AL. Toddler skills predict moderate-to-late preterm born children’s cognition and behaviour at 6 years of age. PLoS ONE. 2019;14(11):e0223690. 31693682 10.1371/journal.pone.0223690 PMC6834277

[23] Marchman VA, et al. Speed of language comprehension at 18 months predicts school-relevant outcomes at 54 months in children born preterm. J Dev Behav Pediatrics: JDBP. 2018;39(3):246. 29309294 10.1097/DBP.0000000000000541 PMC5866178

[24] Schoon I, et al. Childhood language skills and adult literacy: a 29-year follow-up study. Pediatrics. 2010;125(3):e459–66. 20142287 10.1542/peds.2008-2111

[25] Fernald A, Marchman VA. Language learning in infancy, in *Handbook of psycholinguistics*. Elsevier; 2006. pp. 1027–71.

