# Supplementary material for: The relationship between the ages and stages questionnaire, 3rd edition scores in early childhood and future cognitive abilities in young Nepalese children
Source: BMC Pediatr. 2024 Oct 10;24:642. doi: 10.1186/s12887-024-05112-3 (PMC11465539; doi:10.1186/s12887-024-05112-3)
Supplement: Supplementary file 1 — Supplementary Material 1 [file 12887_2024_5112_MOESM1_ESM.docx]

**Supplementary material**

| **Supplementary table 1. Key demographic characteristics of a Nepalese child cohort** | | | | |
| --- | --- | --- | --- | --- |
|  | Total cohort  N=600 | Children with data at T1 and T3  N=526 | Children with data at T2 and T3  N=509 | Children with data at T1 and T2  N=539 |
| Age in months, mean (sd) |  |  |  |  |
| T1; 6-11 months | 8.4 (1.8) | 8.3 (1.8) | 8.4 (1.8) | 8.4 (1.8) |
| T2; 18-23 months | 20.0 (1.8) | 20.0 (1.8) | 20.0 (1.8) | 20.0 (1.8) |
| T3; 42-47 months | 43.9 (1.9) | 43.9 (1.9) | 43.9 (1.9) | 43.9 (1.9) |
| Female | 291 (48.5%) | 258 (49.1%) | 250 (49.1%) | 264 (49.0%) |
| Birth weight in grams, mean (sd) | 3040.1 (1413.4) | 2984.6 (1300.7) | 3006.7 (1355.7) | 3032.4 (1421.7) |
| Children stunted at T1; 6-11 months | 162 (27.0%) | 147 (28.0%) | 141 (27.8%) | 150 (27.9) |
| Maternal age in years at T1, mean (sd) | 27.3 (4.7) | 27.7 (4.6) | 27.7 (4.6) | 27.6 (4.6) |
| Maternal literacy |  |  |  |  |
| Illiterate up to grade 5 | 223 (37.2%) | 188 (35.7%) | 186 (36.5%) | 198 (36.7%) |
| Grade 5 to High School | 261 (43.5%) | 234 (44.5%) | 224 (44.0%) | 234 (43.4%) |
| Bachelor and above | 116 (19.3%) | 104 (19.8%) | 99 (19.5%) | 107 (19.9%) |
| Paternal literacy |  |  |  |  |
| Illiterate up to grade 5 | 212 (35.3%) | 184 (35.0%) | 180 (35.4%) | 190 (35.3%) |
| Grade 5 to high School | 280 (46.7%) | 248 (47.2%) | 236 (46.5%) | 251 (46.7%) |
| Bachelor and above | 108 (18.0%) | 93 (17.7%) | 92 (18.1%) | 97 (19.0%) |
| All figures are numbers (%) if not otherwise specified |  | | | |
